# Relationships Between Biological Heavy Metals and Breast Cancer: A Systematic Review and Meta-Analysis

**DOI:** 10.3389/fnut.2022.838762

**Published:** 2022-06-06

**Authors:** Lin Liu, Jie Chen, Chang Liu, Yuxuan Luo, Jiayun Chen, Yuanyuan Fu, Yajie Xu, Haili Wu, Xue Li, Hui Wang

**Affiliations:** ^1^State Key Laboratory of Oncogenes and Related Genes, Center for Single-Cell Omics, School of Public Health, Shanghai Jiao Tong University School of Medicine, Shanghai, China; ^2^College of Life Science, Shanxi University, Taiyuan, China

**Keywords:** breast cancer, copper, cadmium, lead, manganese

## Abstract

**Introduction:**

Heavy metals were classified as essential, probably essential, and potentially toxic in the general population. Until now, it has been reported inconsistently on the association between heavy metals and BC. In this meta-analysis, we aimed to assess the association between heavy metals and BC and review the potential mechanisms systematically.

**Methods:**

We searched for epidemiological studies in English about the association between heavy metals and BC published before September 2020 in PubMed, Web of Science, and Embase databases. In total 36 studies, comprising 4,151 individuals from five continents around the world were identified and included.

**Results:**

In all biological specimens, Cu, Cd, and Pb concentrations were higher, but Zn and Mn concentrations were lower in patients with BC than in non-BC participants [SMD (95% CIs): 0.62 (0.12, 1.12); 1.64 (0.76, 2.52); 2.03 (0.11, 3.95); −1.40 (−1.96, −0.85); −2.26 (−3.39, −1.13); *p* = 0.01, 0.0003, 0.04, <0.0001, <0.0001]. Specifically, higher plasma or serum Cu and Cd, as well as lower Zn and Mn, were found in cases [SMD (95% CIs): 0.98 (0.36, 1.60); 2.55 (1.16, 3.94); −1.53 (−2.28, −0.78); −2.40 (−3.69, −1.10); *p* = 0.002, 0.0003, <0.0001, 0.0003]; in hair, only lower Zn was observed [SMD (95% CIs): −2.12 (−3.55, −0.68); *p* = 0.0004]. Furthermore, the status of trace elements probably needs to be re-explored, particularly in BC. More prospective studies, randomized clinical trials, and specific pathogenic studies are needed to prevent BC. The main mechanisms underlying above-mentioned findings are comprehensively reviewed.

**Conclusion:**

For BC, this review identified the current knowledge gaps which we currently have in understanding the impact of different heavy metals on BC.

**Systematic Review Registration:**

www.crd.york.ac.uk/prospero/display_record.php?ID=CRD42020176934, identifier: CRD42020176934.

## Introduction

Heavy metals exist in the Earth's crust as the natural ingredients and are present in all aspects of the environment, including the air, water, soil, and plants (1). Human beings absorb heavy metals mainly from crops, vegetables, water, and sediments (1–4). Heavy metals are difficult to biodegrade *in vivo*, and an overload of these metals in the body can cause vomiting, stomach irritation, hair loss, cardiovascular disease, diabetes, leukemia, and other diseases (5–8). On the other hand, the lack of several special heavy metals leads to cardiovascular diseases, central nervous system diseases, and others (9, 10). The impact of heavy metals on human health has become an important public health problem; either excess or deficient content of heavy metals leads to a variety of potential health hazards.

As the most universal malignant disease of women worldwide, the incidence of cases of breast cancer (BC) is expected to increase by more than 46% by 2040 (11). In addition to genetic factors, smoking, and lifestyle factors, which are known to be related to raising the BC risk (12), in recent years, *in vivo* metabolism of several heavy metals has been found to have something to do with BC (13). For example, cadmium (Cd) has been reported to mimic estrogenic effects to promote the development of BC (14). The ROS pathway mediated the effects of copper (Cu), zinc (Zn), and manganese (Mn) on the occurrence and development of BC (15). However, the published results are varied in different biological samples. Thus, it is not clear which heavy metals and which kind of biological samples can be used as predictors of BC incidence. To this end, it is necessary to clarify the reliable relationships and underlying mechanisms of heavy metals on BC.

In the human body, the influence of the environment, nutritional status, or metabolism of heavy metals could be reflected by detecting the biomarkers present in the blood, tissue, skin, and nails (16–20). In this review, population, exposure, comparator, and outcome (“PECO”) approach has been used to analyze the relationship between different heavy metals and BC (21). The question below was answered through PECO: What are the relationships between heavy metal concentration in plasma or serum, tissue, skin, and nails and risk of BC? We then systematically summarized the existing relevant mechanisms. The systematic review and meta-analysis were conducted in accordance with the COSTER recommendations (22).

## Methods

PubMed, Web of Science, and Embase databases were searched to confirm studies published up until September 2020 on the relationship between heavy metal concentration and BC. The research question was generated by merging keywords representing the exposure and outcome components according to the PECO guideline (21). Detailed study selection and data analysis: refer to Supplementary File 1. The following search keywords were used in the search strategy: “heavy metals” or “trace elements” combined with “breast cancer” or “mammary carcinoma.” Besides, we searched all results in the reported reviews and all relevant meta-analyses. A protocol of the systematic review was registered a priori in the PROSPERO register (International Prospective Register of Systematic Reviews), number CRD42020176934 https://www.crd.york.ac.uk/prospero/. The quality of included studies was assessed by Newcastle–Ottawa scale (NOS) and Critical Appraisal Skills Program (CASP) checklist.

### Study Selection

Qualified studies had to accord with the following standards: (1) the population (P) was restricted to the general population; (2) exposure (E) to heavy metals was estimated by long-term exposure biomarkers, i.e., evaluation of plasma/serum/tissue/hair/nail concentration; (3) the comparator (C) was specified for including individuals without BC; (4) the outcome (O) was BC prevalence; (5) studies of human beings were included; and (6) the studies that were available in the English language. The exclusive criteria were as follows: (1) animal studies; (2) *in vitro* or laboratory studies; (3) studies that did not present original data; (4) studies that evaluated heavy metals not in the human body; (5) studies conducted in the nuclear radiation areas; and (6) studies that did not report the heavy metal content in the BC cases and healthy participants.

### Data Collection

Data were collected from the confirmed studies according to a standardized procedure. The information picked up included first author, study design, year of publication, geographic area, sample size, age, and heavy metals concentration.

### Data Analysis

The quality of included studies was assessed by NOS and CASP checklist (23, 24) (Supplementary Tables 1, 2). The study quality assessed by NOS was as follows: low quality: 0–3; moderate quality: 4–6; high quality: 7–9 (25). A number of two authors double-checked and completed all the above process independently. The extracted data were used for meta-analyses to acquire the standardized mean differences (SMDs) and 95% confidence intervals (95% CIs). The Q-test and *I*^2^ tests were used to examined the heterogeneity among studies. In case the *I*^2^> 50% or the *p* < 0.05, heterogeneity was considered in the meta-analysis. The SMDs were computed by random-effects model with heterogeneity. Subgroup analyses were also conducted stratified by geographic background and source of the biological samples including plasma or serum, hair, tissue, and toenails. Potential publication bias of studies was estimated by funnel plots and Egger test (26). Sensitivity analysis was carried out to investigate the constancy of the results; for this, each included study was excluded, one at a time, and the meta-analysis was re-run with the exclusion of each study. All the statistical analyses were conducted by Review Manager 5.3. The world maps on the association between heavy metals and BC were conducted by PyCharm 2021.3.3 (Community Edition).

## Results

**Figure 1:**
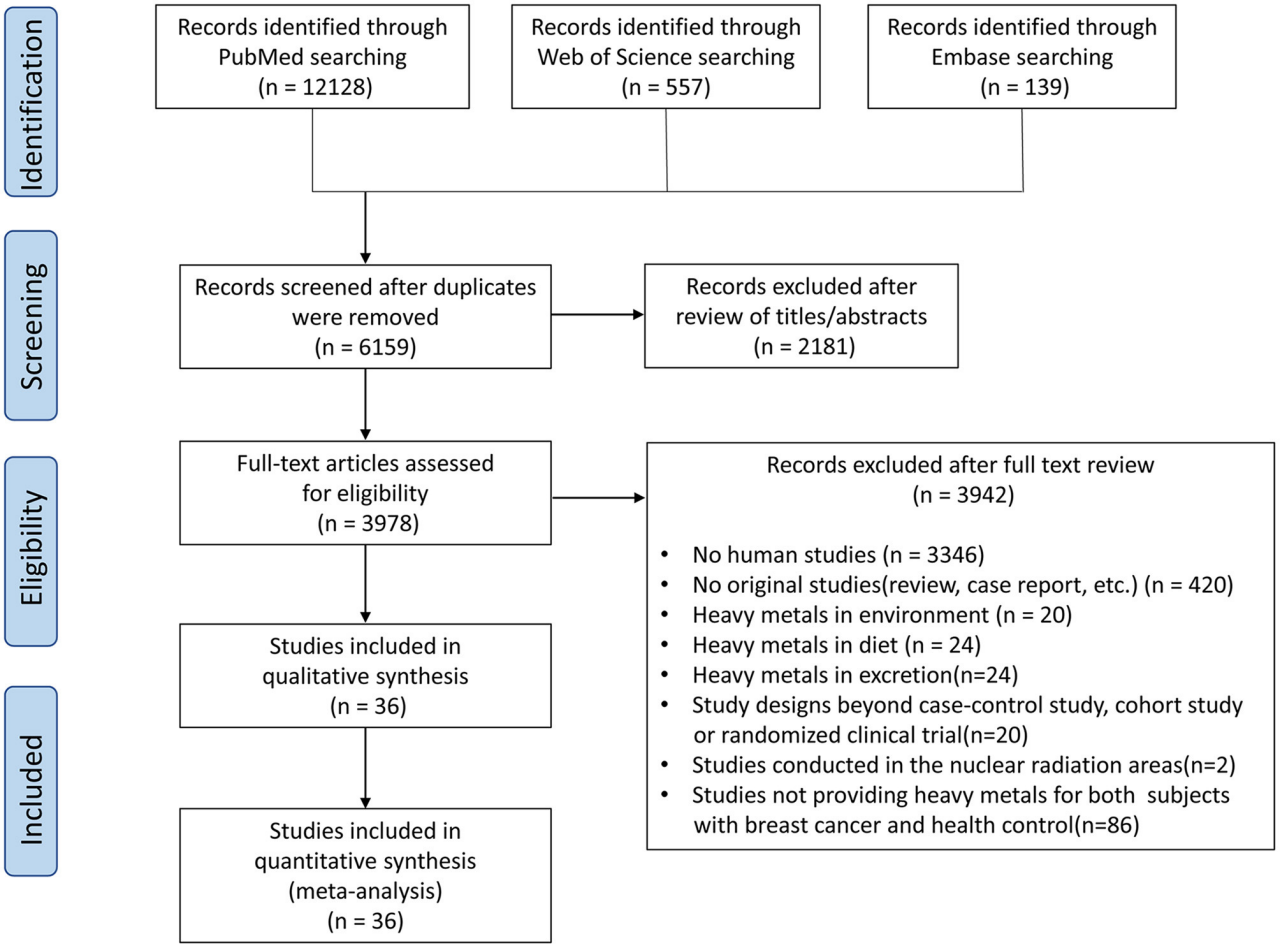
Working flowchart for the systematic selection of studies for this meta-analysis.

The literature search produced 12,824 initial studies, containing 12,128 studies from PubMed, 557 studies from Web of Science, and 139 studies from Embase (Figure 1). After a detailed review of records, 36 case–control studies (including two nested case–control studies) with a total of 4,151 individuals (1,996 cases and 2,155 non-BC participants) were selected for this meta-analysis. Supplementary Tables 1, 2 show the quality assessment of included articles using the NOS and CASP checklist. Based on NOS, all the studies were evaluated with 5 or more points, which means that no studies with low quality were included. Among them, the quality scores of two studies were 7. The others' quality scores were between 5 and 7. Supplementary Table 3 gives detailed characteristics of the studies used for the meta-analysis. A total of 19 studies were conducted in Asia (27–45), 12 in Europe (46–57), 2 in Africa (19, 58), 2 in South America (59, 60), and 1 in North America (61). There are nine heavy metals that are categorized into three groups according to the WHO classification (62): essential trace elements — Cu, chromium (Cr), cobalt (Co), iron (Fe), Zn; probably essential trace elements — Mn, nickel (Ni); potentially toxic trace elements — Cd, lead/plumbum (Pb).

### Meta-Analysis of the Associations Between Essential Trace Elements According to the WHO Definition and BC

#### Copper and Breast Cancer

**Figure 2:**
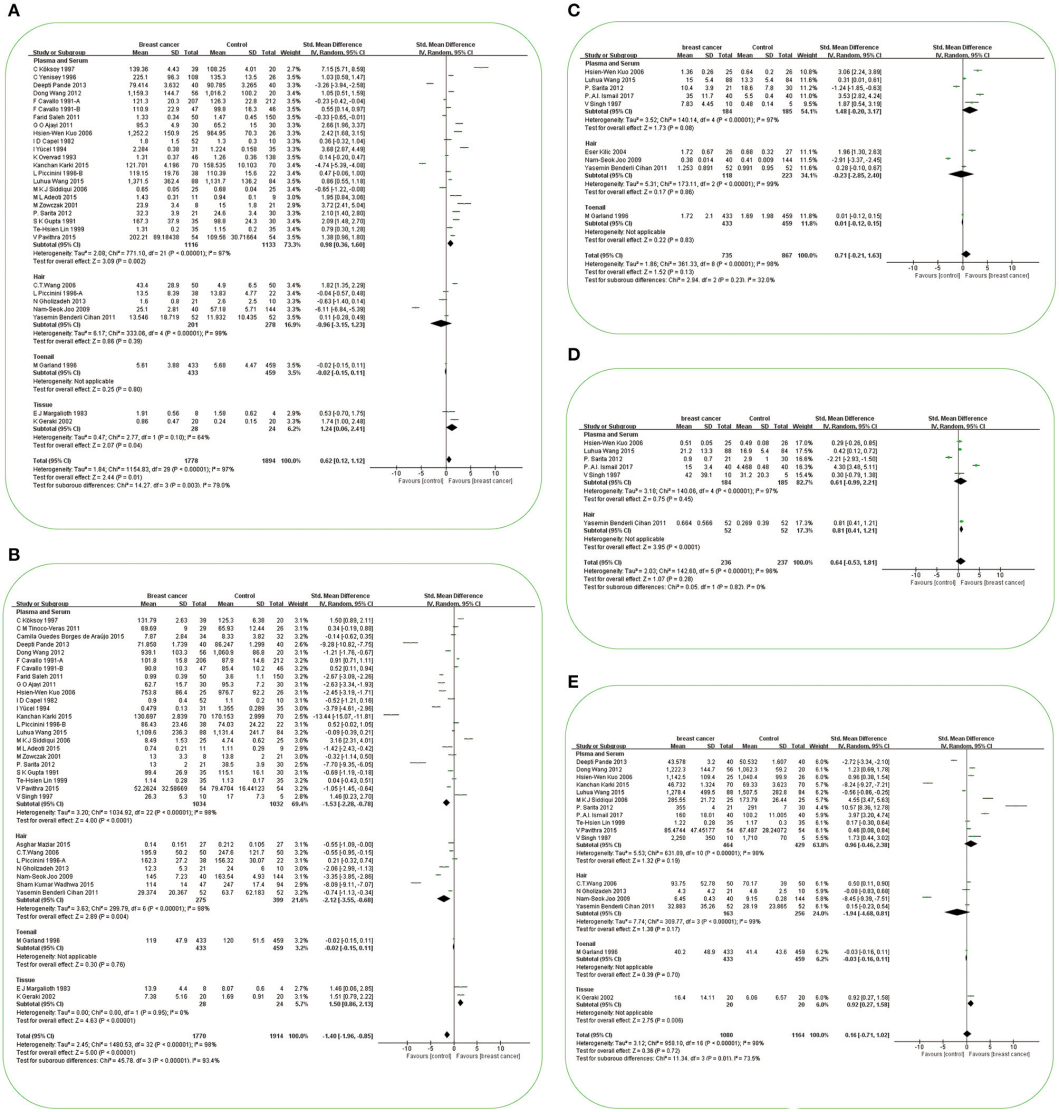
Forest plot of studies of essential trace elements in subjects with breast cancer vs. non-breast cancer controls. **(A)** Forest plot of studies of Cu levels in subjects with breast cancer vs. non-breast cancer controls. **(B)** Forest plot of studies of Zn levels in subjects with breast cancer vs. non-breast cancer controls. **(C)** Forest plot of studies of Cr levels in subjects with breast cancer vs. non-breast cancer controls. **(D)** Forest plot of studies of Co levels in subjects with breast cancer vs. non-breast cancer controls. **(E)** Forest plot of studies of Fe levels in subjects with breast cancer vs. non-breast cancer controls. The standard mean differences (SMD) and 95% confidence intervals (CIs) were calculated using the random-effects model. Cu, copper; Zn, zinc; Cr, chromium; Co, cobalt; Fe, iron.

**Figure 3:**
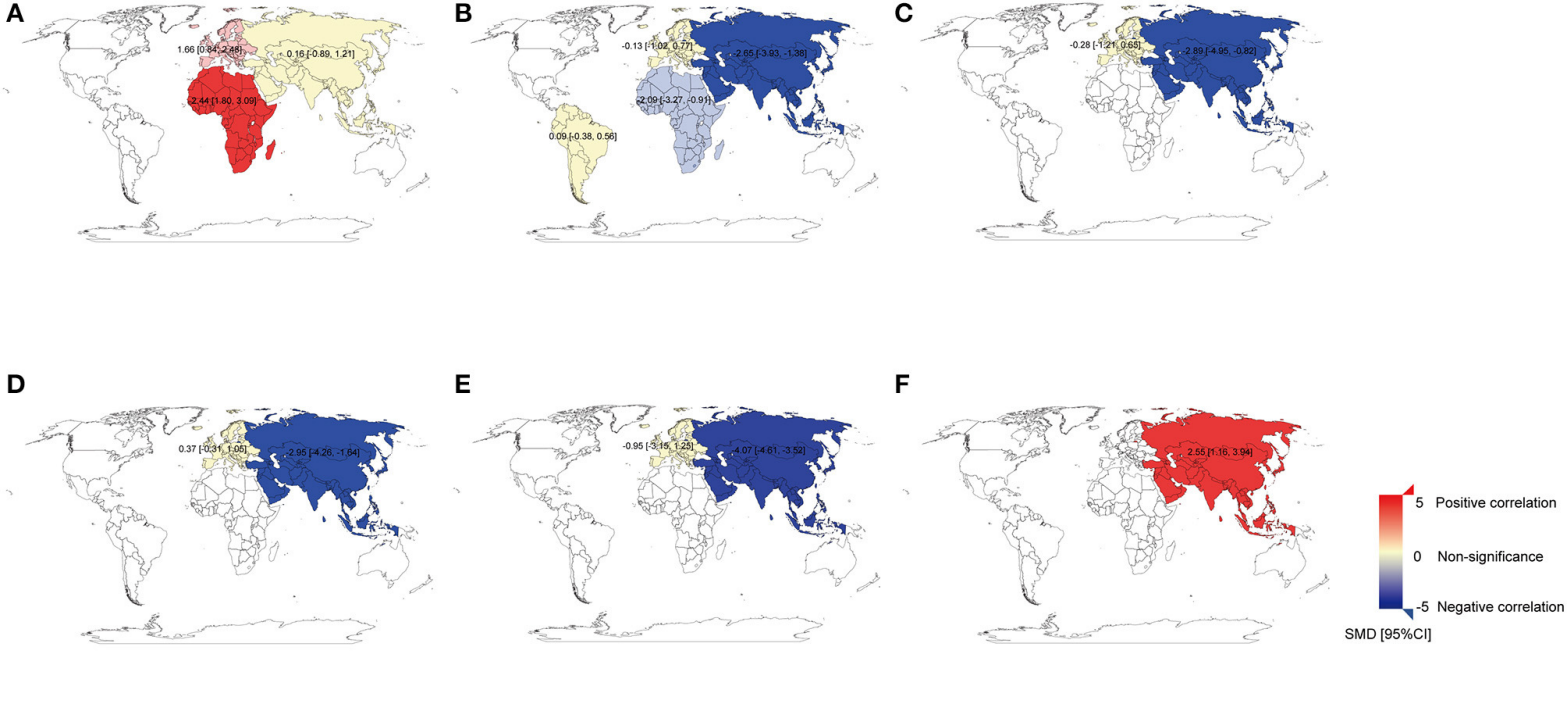
The world map for the associations between heavy metals and breast cancer. **(A)** The world map for the association between Cu and breast cancer in plasma/serum. **(B)** The world map for the association between Zn and breast cancer in plasma/serum. **(C)** The world map for the association between Zn and breast cancer in hair. **(D)** The world map for the association between Mn and breast cancer in plasma/serum. **(E)** The world map for the association between Mn and breast cancer in hair. **(F)** The world map for the association between Cd and breast cancer in plasma/serum. The standard mean differences (SMD) and 95% confidence intervals (CIs) were calculated using the random-effects model and remarked in the maps by regions. Cu, copper; Zn, zinc; Mn, manganese; Cd, cadmium.

A total of 30 studies investigated the differences in Cu concentration between patients with BC and non-BC participants (Figure 2A). In plasma/serum and tissue, Cu concentration in patients with BC was significantly higher than those in non-BC participants. In hair and toenails, the relationship was non-significant (*p* > 0.05). In Africa and Europe, patients with BC have a higher Cu concentration in plasma/serum than in non-BC participants [SMD (95% CIs): 2.44 (1.80, 3.09), 1.66 (0.84, 2.48); (*I*^2^ = 11%, *I*^2^ = 96%)]. In Asia, it was found non-significance in plasma/serum [SMD (95% CIs): 0.16 (−0.89, 1.21), *I*^2^ = 98%] (Figure 3A). No significant difference was found in hair Cu concentration between patients with BC and non-BC in Asia and Europe. The total number of studies on toenail and tissue was limited to be analyzed by region further. No significant publication bias was found for all the studies (*p* ${}_{\text{Egge}{\text{r}}^{\text{′}}\text{stest}}$ = 0.21).

#### Zinc and Breast Cancer

A total of 33 studies investigated the differences in Zn concentration between patients with BC and non-BC participants (Figure 2B). In plasma/serum and hair, Zn concentration in patients with BC was significantly lower than in non-BC participants, but it was reversed in tissue. In toenails, no significant difference was found between BC and non-BC participants (*p* > 0.05). In Africa and Asia, BC patients have a lower Zn concentration in plasma/serum than in non-BC participants [SMD (95% CIs): −2.09 (−3.27, −0.91); −2.65 (−3.93, −1.38); *I*^2^ = 73%, 98%] (Figure 3B). In Asia, it was also found to be significant in hair [SMD (95% CIs):−2.89 (−4.95, −0.82); *I*^2^ = 98%] (Figure 3C). But it was non-significant for the results on plasma/serum in European and South American and hair in Europe (Figures 3B,C) (all *P* > 0.05). The total number of studies on toenails was limited to be analyzed by region further. Publication bias was found in all the studies (*p* ${}_{\text{Egge}{\text{r}}^{\text{′}}\text{stest}}$ = 0.0053).

#### Chromium and Breast Cancer

A total of nine studies investigated the differences in Cr concentration between patients with BC and non-BC participants (Figure 2C). In plasma/serum, hair, and toenails, there were no significant differences in Cr concentration between BC and non-BC participants. In Asia, non-significance of Cr concentration in plasma/serum between the BC and non-BC participants was found. But patients with BC in Asia had a lower Cr concentration in hair than non-BC participants [SMD (95% CIs): −2.91 (−3.37, −2.45)]. In Europe, Cr concentration was found to be non-significant in hair [SMD (95% CIs): 1.10 (−0.55, 1.75), *I*^2^ = 95%]. The total number of studies on toenails was limited to be analyzed by region further. No significant publication bias was found for all the studies (*p* ${}_{\text{Egge}{\text{r}}^{\text{′}}\text{stest}}$ = 0.46).

#### Cobalt and Breast Cancer

A total of six studies investigated the differences in Co concentration between patients with BC and non-BC participants (Figure 2D). In plasma/serum, there was no significant difference in Co concentration between the two groups of participants. In hair, Co concentration in patients with BC was significantly higher than those in non-BC participants. In Asia, Co concentration was found to be non-significant in plasma/serum. The total number of studies on hair was limited to be analyzed by region further. No significant publication bias was found for all the studies (*p* ${}_{\text{Egge}{\text{r}}^{\text{′}}\text{stest}}$ = 0.87).

#### Iron and Breast Cancer

A total of 17 studies investigated the differences in Fe concentration between patients with BC and non-BC participants (Figure 2E). In plasma/serum, hair, and toenails, there was no significant difference in Fe concentration between BC and non-BC participants. In tissue, Fe concentration in patients with BC was significantly higher than those in non-BC participants. In Asia, Fe concentration was found to be non-significant in plasma/serum and hair. In Europe, Fe concentration was non-significant in hair. The total number of studies on toenails was limited to be analyzed by region further. No significant publication bias was found for all the studies (*p* ${}_{\text{Egge}{\text{r}}^{\text{′}}\text{stest}}$ = 0.92).

### Meta-Analysis of the Associations Between Probably Essential Trace Elements According to the WHO Definition and BC

#### Nickel and Breast Cancer

**Figure 4:**
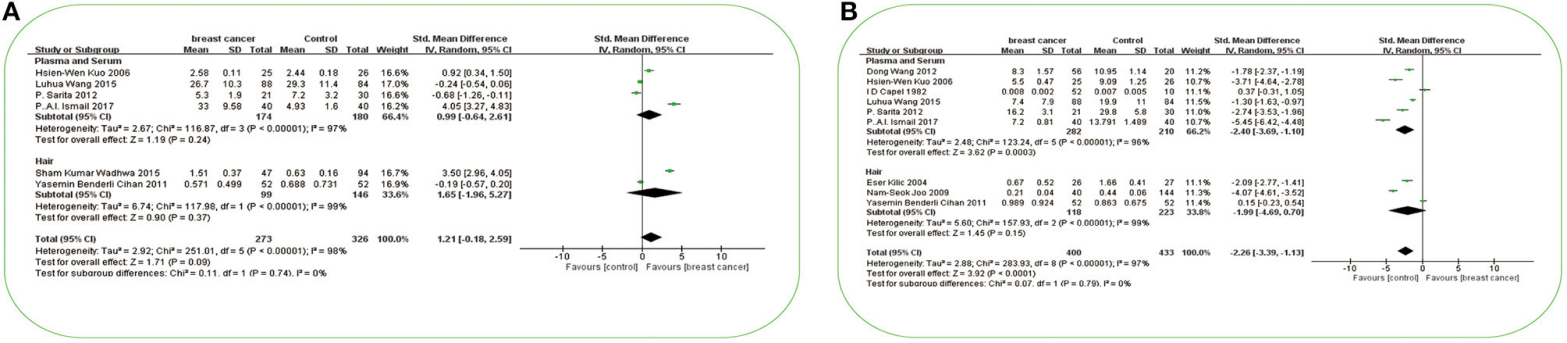
Forest plot of studies of possible essential trace elements in subjects with breast cancer vs. non-breast cancer controls. **(A)** Forest plot of studies of Ni levels in subjects with breast cancer vs. non-breast cancer controls. **(B)** Forest plot of studies of Mn levels in subjects with breast cancer vs. non-breast cancer controls. The standard mean differences (SMD) and 95% confidence intervals (CIs) were calculated using the random-effects model. Ni, nickel; Mn, manganese.

A total of six studies investigated the differences in Ni concentration between patients with BC and non-BC participants (Figure 4A). In plasma/serum and hair, there was no significant difference in Ni concentration between BC and non-BC participants. In Asia, Ni concentration was non-significant in plasma/serum. The total number of studies on hair was limited to be analyzed by region further. No significant publication bias was found for all the studies (*p* ${}_{\text{Egge}{\text{r}}^{\text{′}}\text{stest}}$ = 0.14).

#### Manganese and Breast Cancer

A total of nine studies investigated the differences in Mn concentration between patients with BC and non-BC participants (Figure 4B). In plasma/serum, Mn concentration in patients with BC was significantly lower than those in non-BC participants. In hair, there was no significant difference in Mn concentration between BC and non-BC participants. In Asia, Mn concentration in plasma/serum and hair in patients with BC was significantly lower than those in non-BC participants [SMD (95% CIs): −2.95 (−4.26, −1.64), −4.07 (−4.61, −3.52); *I*^2^ = 95%]. In Europe, Mn concentration was non-significant in plasma/serum and hair (Figures 3D,E) (all *p* > 0.05). No significant publication bias was found for all the studies (*p* ${}_{\text{Egge}{\text{r}}^{\text{′}}\text{stest}}$ = 0.10).

### Meta-Analysis of Associations Between Potentially Toxic Trace Elements According to the WHO Definition and BC

#### Cadmium and Breast Cancer

**Figure 5:**
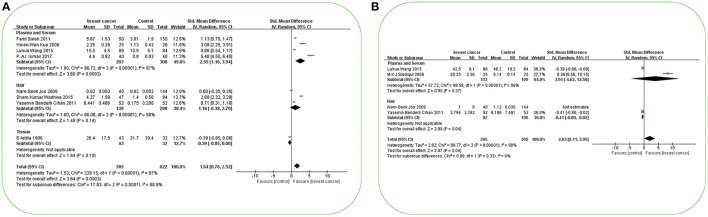
Forest plot of studies of potential toxic trace elements in subjects with breast cancer vs. non-breast cancer controls. **(A)** Forest plot of studies of Cd levels in subjects with breast cancer vs. non-breast cancer controls. **(B)** Forest plot of studies of Pb levels in subjects with breast cancer vs. non-breast cancer controls. The standard mean differences (SMD) and 95% confidence intervals (CIs) were calculated using the random-effects model. Cd, cadmium; Pb, lead.

A total of eight studies investigated the differences in Cd concentration between patients with BC and non-BC participants (Figure 5A). In plasma/serum and hair, Cd concentration in patients with BC was significantly higher than those in non-BC participants. In Asia, Cd concentration in plasma or serum in patients with BC was significantly higher than it in non-BC participants [SMD (95% CIs): 2.55(1.16, 3.94); *I*^2^ = 97%] (Figure 3F). In Europe, it was found to be significant in hair [SMD (95% CIs): 0.71(0.31, 1.10)]. The total number of studies on hair and tissue was limited to be analyzed by region further. In the publication bias test, a slight publication bias was found using Egger's test (*p* = 0.06).

#### Lead and Breast Cancer

A total of four studies investigated the differences in Pb concentration between patients with BC and non-BC participants (Figure 5B). In plasma/serum, there was no significant difference in Pb concentration between BC and non-BC participants. In hair, Pb concentration in patients with BC was significantly lower than those in non-BC participants. In Asia, Pb concentration was non-significant in plasma and serum. The total number of studies on hair was limited to be analyzed by region further. No significant publication bias was found for all the studies (*p* ${}_{\text{Egge}{\text{r}}^{\text{′}}\text{stest}}$ = 0.70).

**Figure 6:**
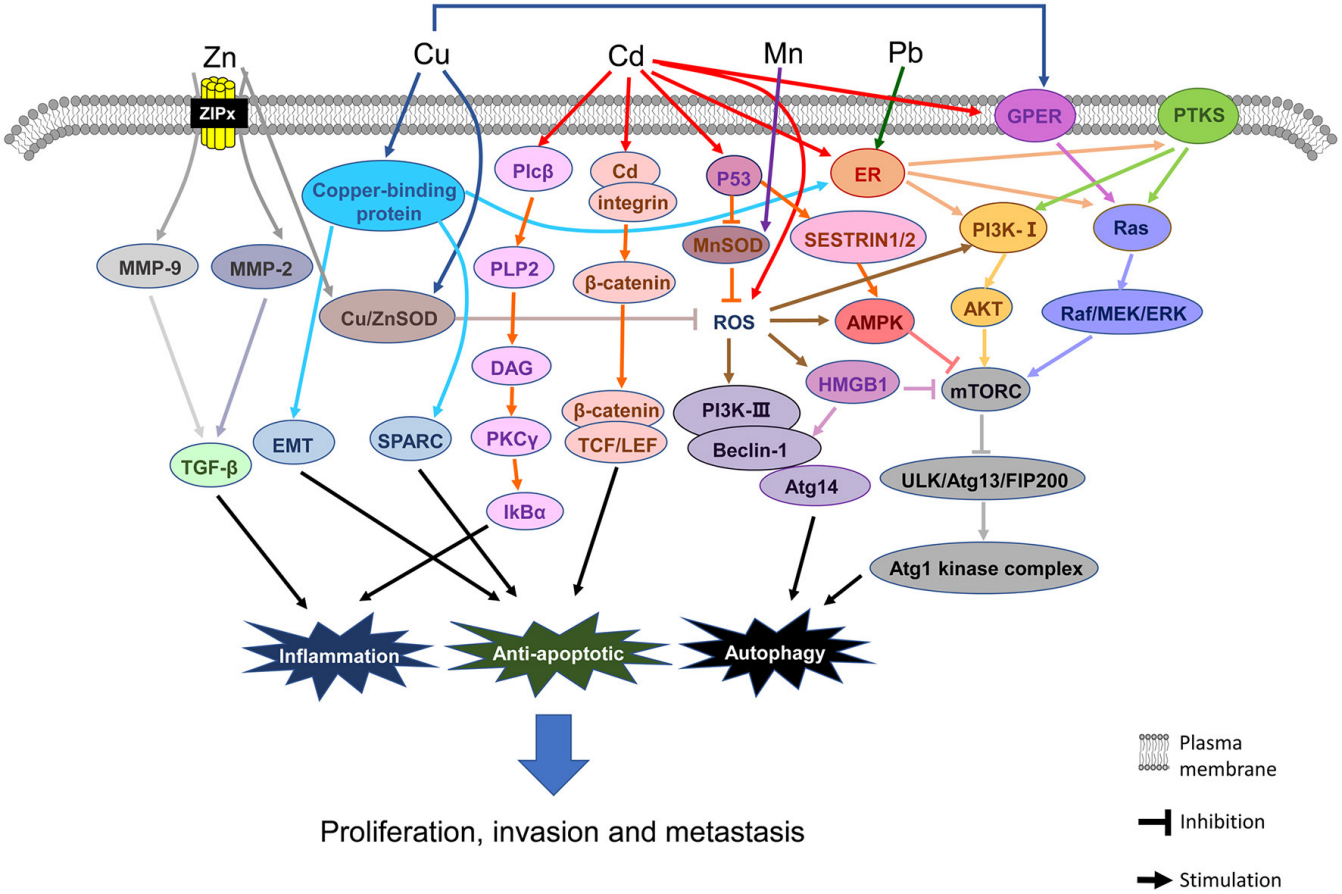
Signaling pathways involved in the associations between heavy metals and breast cancer.

In addition, the symmetry of the funnel plots also indicates less evidence of publication bias. However, this bias could not be completely ruled out due to the limited number of publications. Figure 6 shows the details of the mechanisms underlying the associations between BC and Cu, Cd, Pb, Zn, and Mn. Finally, the evidence for the association between heavy metals and BC was low, due to the case–control studies included and the publication bias of studies about Zn and Cd (Supplementary Table 4).

## Discussion

Across all biological specimens, we discovered that Cu, Cd, and Pb concentrations in patients with BC were significantly higher than those in non-BC participants, but Zn and Mn concentrations were significantly lower than them (Supplementary Table 4). In plasma/serum, Cu and Cd concentration in patients with BC was significantly higher than those in non-BC participants, but Zn and Mn concentrations were significantly lower than them. In hair specimens, only Zn concentration in patients with BC was significantly lower than those in non-BC participants. Furthermore, the subgroup analyses indicated variation in findings across different ethnic groups. The detailed mechanisms have been described and suggest that Cu, Cd, Pb, Zn, and Mn may eventually lead to BC through different molecular modifications.

### Associations Between Essential Trace Elements According to the WHO Definition and BC

#### Copper and Breast Cancer

Cu is one of the essential trace elements for the general population. It exists in two forms, Cu^+^ and Cu^2+^. Cu in the diet is in the bivalent form, which is restored to monovalent state by related enzymes before being transported into cells (63). The delicate *in vivo* balance of Cu is preserved by ATP7A and ATP7B as two Cu-transporting adenosine triphosphatases (ATPases) bound to the membrane (63). Cu in the reduced state is bound to its chaperone and it is transferred to ATP7A and ATP7B through antioxidant-1 (ATOX1) as a chaperone (64). The small intestine is the site where most dietary Cu is absorbed, and Cu flows out in the bile from the liver (65).

In this meta-analysis, across various specimens from human beings, we found a statistically significant difference in Cu concentration between patients with BC and non-BC participants. In plasma/serum and tissue, Cu concentration in patients with BC was significantly higher than those in non-BC participants; however, no significant differences were obtained between cases and non-BC participants in hair and toenail specimens. There are several possible reasons for the variation in findings as a function of the sample source. First, of the human specimens, blood and plasma/serum are the strongest proof specimens for diagnosis of deficiency or exposure to Cu (66), but hair Cu content can be influenced by dyeing, bleaching, shampoos, and the gap to the scalp; thus, hair is not appropriate for evaluating Cu concentration (67). The small quantity of studies included in this meta-analysis on Cu in tissue and toenails is another possible reason. In the subgroup analysis by region, the result showed that the Cu concentration in plasma/serum of patients with BC in Africa and Europe was significantly higher than those in non-BC participants; however, there was no significant difference in Cu concentration between Asian cases and non-BC participants. The reason for this difference as a function of ethnicity may be that the obesity rate is lower in Asia than in Africa and Europe (68). The content of Cu in serum is known to be elevated in obesities (69, 70). Along with serum Cu concentration, the ceruloplasmin, as the body's main Cu carrier, is overexpressed in adipose tissue and obesity-related cancer cells (71). It should be noted that there is currently no data on Cu from patients with BC in Australia and America. With respect to the findings from Africa, enrichment of Cu has been observed in the natural environment in Africa, such as in plants, soil, and rivers (72–74), which is partly caused by mining activity, disposal of E-waste, and so on (75–78).

Figure 6 shows the main mechanistic pathways from *in vivo/vitro* experiments. The most important factors and pathways involved in Cu elevation and the pathogenesis of BC are copper-binding protein, G-protein estrogen receptor (GPER), and reactive oxygen species (ROS). ① For copper-binding protein, the LOX-like (LOXL) family of proteins, mediator of cell motility 1 (MEMO1), and ATOX1 are the three major proteins related to BC (79). It has been shown that the LOXL family of proteins promotes the invasion or metastasis phenotype of BC cells *via* the epithelial-mesenchymal transition (EMT) and secreted protein acidic and rich in cysteine (SPARC) pathways (80, 81). MEMO1 promotes tumor cell migration and metastasis *via* EMT-related pathways, which are obtained from BC cells (82). MEMO1 also controls the subcellular localization and phosphorylation of the estrogen receptor (ERα) and downstream function of ErbB2/ER or IGFIR/ER, thus activating the MAPK and PI3K signaling pathways and promoting the migration and/or proliferation of BC cells (83, 84). ATOX1 may also act on the migration of BC cells unknowingly (85). ② Cu also stimulates estrogenic GPER signaling transduction, inducing expression of the Raf/MEK/ERK signaling pathways and activating the downstream pathway of mTOR, finally leading to angiogenesis and tumor growth in BC cells (86). ③ Cu is an accessory factor of copper-zinc superoxide dismutase (CuZnSOD), but its activity is influenced by the concentration of Zn (87). Furthermore, Cu chaperone (CCS) (88) and vascular endothelial growth factor receptor 2^+^ (VEGFR2^+^) endothelial progenitor cells (EPCs) are the signaling pathways for Cu to promote BC *in vivo* (89).

#### Zinc and Breast Cancer

Zn is one of the essential trace elements for the general population. It exists in organisms in the form of redox-inert Zn^2+^ (90). Zn is mainly distributed in the liver, and the gastrointestinal tract is the main organ for Zn absorption and excretion (90). The homeostasis of Zn in cells is monitored by Zn transporters (ZnT) regulating the outflow of Zn and by Zn importers (ZIP) regulating the inflow of Zn (91, 92).

In this meta-analysis, Zn concentration in plasma/serum and hair from patients with BC was significantly lower than those in non-BC participants; however, in tissue, Zn concentration in patients with BC was significantly higher than those in non-BC participants, but the total number of the studies for the analysis is not large enough. There are several possible reasons for the similar results for plasma/serum and hair specimens. First, plasma Zn is the most widely applied and widely accepted biomarker of Zn status (20). Furthermore, Zn is a structural component of the hair matrix formed in hair follicles, and the Zn concentration in hair reflects, to some extent, the availability of Zn from the blood supply during hair growth (93). Besides, there are also several possible reasons for the different results between tissue and plasma/serum or hair. Plasma Zn can be used as a biomarker of Zn deficiency to assess the Zn nutritional conditions of a population (94). Metallothionein (MT)/thionein (T) couple is a homeostatic system of Zn. When the nutrition of Zn in the body is sufficient, Zn is used to guide the T synthesis and result in the MT formation; when the amount of available Zn is low, Zn is released from MT (95, 96). Therefore, the relationship between Zn and BC may be U-shaped; excess or deficiency of Zn may have adverse effects (97). On the one hand, when the body has too little Zn, the absorption and utilization of Zn are reduced, CuZnSOD synthesis is reduced, and ROS is increased (98). On the other hand, if there is too much Zn, it will accumulate in the body.

Figure 6 shows the main mechanistic pathways from *in vivo/in vitro* experiments. The most important factors and pathways involved in Zn deficiency and the pathogenesis of BC are MMPs and ROS, respectively. Once Zn enters a cell, there is a decrease in Zn in the blood and the Zn compartmentalization promotes invasion, metastasis, and angiogenesis of tumors, which is mediated by matrix metalloproteinases, especially MMP-2 and MMP-9 (99). Zn keeps the structure of the effective zone of CuZnSOD, which can effectively suppress cancer cell growth (98). If the Zn concentration in the blood is low, there may not be enough Zn to maintain the structure of the active site of CuZnSOD to slow down the cancer cell growth, thus boosting cancer cell growth rapidly.

In the subgroup analysis by region, we found that Zn concentration in the plasma/serum of patients with BC in Africa and Asia was significantly lower than those in non-BC participants; it was also found to be significant in hair in Asia; no significant differences between cases and non-BC participants were found in samples from Europe and South America. The reason for these differences may attribute to the dietary shortage of Zn in Africa and Asia, especially in South Asia, South East Asia, and sub-Saharan Africa (100, 101). In addition, rs10822013 on the chromosome at 10q21.2 in the zinc finger protein 365 (ZNF365) gene is a genetic risk variant for all four stages of BC among East-Asian women (102). In this study, there were too few studies of Zn in hair specimens from European samples and in toenail specimens to enable further analysis.

#### Chromium and Breast Cancer

Cr is one of the essential trace elements for the general population. In the environment, Cr has a variety of oxidation valence states: Cr^0^ (elemental chromium), Cr^1+^, Cr^2+^, Cr^3+^, Cr^4+^, Cr^5+^, and Cr^6+^, among which Cr^3+^ is the most stable, followed by Cr^6+^ (103). The main source of oral Cr in non-occupational populations is oral intake (103). *In vivo*, Cr^6+^ can be conveyed into cells *via* anion transporters and reduced to Cr^3+^ through a series of metabolic reductions (104, 105). Cr^3+^ is mainly excreted through the kidneys (106).

In this meta-analysis, we only found a positive result in hair specimens from one Asian study, but due to the small sample size, this result requires further verification. The studies of hair, toenail, and plasma/serum specimens showed no significant differences between cases and non-BC participants. In this meta-analysis, the form and valence state of Cr were not distinguished, but different valence states lead to different pathogenic effects and mechanisms.

In relation to the mechanism, Cr^3+^ is mainly related to blood glucose homeostasis and is an active component factor of glucose tolerance (GTF), which regulates blood glucose concentration, including carbohydrate and lipid metabolism (107, 108). In addition, Cr^3+^ complexes have been used to treat type 2 diabetes as insulin amplifiers (109). However, for cancer, the carcinogenicity of Cr depends on its valence state. Cr^6+^ was categorized as a class I carcinogen by the International Agency for Research on Cancer (IARC) (110). Cr^6+^ compounds can be introduced into cells through sulfonamides and can then be reduced by a variety of cellular reducers, for example, glutathione (GSH) and ascorbic acid (111). In the process, a spectrum of ROS is generated, which can interact with intermediates and may result in oxidative stress and DNA damage, which are the unstable factors causing mutagenesis (112). The relationships between the concentration of Cr in different valence states and the BC risk and other nutrients need to be explored in detail in various biological specimens.

#### Cobalt and Breast Cancer

Co is one of the essential trace elements for the general population. It mainly exists in two valence states: Co^2+^ and Co^3+^ (113). The most likely source of Co exposure is contaminated food or water (114). Co plays a role in physiological functions in the only known form of Vitamin B_12_ (115). After being taken up by the digestive or respiratory system, some Co is quickly excreted in feces (114). The rest is dispersed throughout the tissues *via* the blood, especially into the liver, kidneys, and bones (114). The absorbed Co slowly leaves the body through the urine (114).

In this meta-analysis, we only found a positive result in hair from one study, but this requires further verification because of the insufficient sample size. We found no differences between cases and non-BC participants in plasma/serum concentration of Co. However, it has been reported that Co induces breast tumors by interfering with signaling, such as estrogen receptor α (ERα) signaling, and simulating hypoxia in angiogenesis and apoptosis, both internally and externally (116, 117). Therefore, Co concentration in BC requires further study.

#### Iron and Breast Cancer

Fe is one of the essential trace elements for the general population. Fe exists as Fe^2+^, Fe^3+^, and Fe^4+^ (90). The main inorganic Fe in the diet is Fe^3+^, which is reduced by ferrireductase duodenal cytochrome b (DCYTB) on the surface of duodenal intestinal cells (90). The resulting Fe^2+^ enters cells through proton-coupled divalent metal transporters (118). In the body, hemoglobin is the main form of Fe; the rest of the Fe in the body is in the form of non-heme enzymes or ferritin in cells. Fe cannot be actively excreted from the human body and is stored in the body for about 10 years (90).

In this meta-analysis, we only found a positive result in tissue from one study, but this result requires replication because of the insufficient sample size. We found no differences in Fe concentration between cases and non-BC participants in plasma/serum, hair, and toenail specimens. However, it has been proved that Fe not only works fundamentally in many pathophysiological functions but also participates in the occurrence of breast tumors by interfering with signalings, such as VEGF, ROS, MAPK, and IL-6/JAK2/STAT3 signaling in animal models (119, 120). Therefore, further research on the effect of Fe concentration in BC is demanded before firm conclusions can be researched.

### Associations Between Probably Essential Trace Elements According to the WHO Definition and BC

#### Manganese and Breast Cancer

Mn is one of the probably essential trace elements for the general population. It is easily oxidized and exists in the form of oxides, carbonates, and silicates in nature (121). In living beings, Mn^2+^ and Mn^3+^ are the most common oxidized states (122). Mn^2+/3+^ is in the effective zone of manganese superoxide dismutase (MnSOD), which is in charge of the ROS detoxification in mitochondria (123). Replacing C with T in the MnSOD gene leads to a change from Val to Ala at the - 9 position of the mitochondrial target sequence (Val-9Ala), thus causing changes to the substructure of MnSOD (124) and affecting the transport of MnSOD into mitochondria (125). The main Mn exposure routes are through dietary intake, skin absorption, and inhalation (126). Once in the bloodstream, Mn is rapidly distributed to various tissues throughout the body *via* blood circulation (121). Most Mn in the body is combined with bile by the liver and excreted in the feces (127).

In this meta-analysis, in plasma/serum, Mn concentration in patients with BC was significantly lower than those in non-BC participants; in hair, there was no significant difference in Mn concentration between the two groups of participants. The possible reason for the different results in the different specimen types is as follows. Mn metabolism and state could be universally evaluated in whole blood or plasma/serum (128), and the Mn concentration in the hair can be confounded by several factors (129). In the subgroup analysis by region, we found that Mn concentration in hair and plasma/serum specimens of patients with BC in Asia was significantly lower than those in non-BC participants; no significant differences were observed in samples from Europe. The reason for this difference may be due to the MnSOD gene polymorphism among Asian women, especially those who eat foods containing less selenium and/or vitamins (130, 131).

Figure 6 shows the major mechanistic pathways described in *in vivo/in vitro* experiments. The most important factor and pathway for Mn elevation and the pathogenesis of BC is ROS signaling. High Mn can make up for the loss of SOD and defend against oxidative stress (15). Like CuZnSOD, when the Mn concentration in blood is low, there is not enough Mn to maintain the structure of the active site of MnSOD, thus promoting rapid cancer cell growth. Moreover, the activity of MnSOD is repressed by p53 at the early stage of BC (132).

#### Nickel and Breast Cancer

Ni is another one of the probably essential trace elements for the general population. It exists in many valence states, Ni^−^-Ni^4+^, among which Ni^2+^ accounts for the largest proportion in the environment and biological systems (133). Ni can get into the human body in three ways – respiratory tract, digestive tract, and skin (134). Once in the body, the absorbed Ni is eliminated from the blood *via* the urinary system, whereas the unabsorbed Ni is excreted *via* the feces (135).

In this meta-analysis, there were no differences between cases and non-BC participants in the plasma/serum and hair specimens nor in the subgroup analyses. Nevertheless, there are some *in vitro* studies describing the mechanism by which Ni could induce BC, particularly in Martin et al., exhibiting that Ni binds to ERα in BC cells and induces cell proliferation *via* mimicking estradiol (116). Thus, future large-scale cohort studies are required in different populations to further investigate the effect of Ni in BC.

### Associations Between Potentially Toxic Trace Elements According to the WHO Definition and BC

#### Cadmium and Breast Cancer

Cd, as one of the potentially toxic trace elements for the general population, is on the list of class 1 carcinogens by the IARC (110). It exists uniquely in the inorganic and divalent state (Cd^2+^) (136). Following exposure, Cd concentration is first highest in the liver. Cd then accumulates in the kidneys and is discharged slowly into the urinary system (137).

In this meta-analysis, in plasma/serum, Cd concentration in patients with BC was found to be significantly higher than those in non-BC participants. In the subgroup analysis by region, we found that Cd concentration in the plasma/serum of patients with BC was significantly higher than those in non-BC participants in Asia. In this meta-analysis, sample test data from China, Iraq, and Kuwait have been included (29, 30, 43, 45). Meanwhile, serious Cd contamination has been found in rice (138), the dominating staple food for people mainly in Asia (139–141). These concomitant findings are of concern for BC prevention and treatment in the future. To be sure, we only found a positive result in hair specimens from one European study, but due to the small sample size, this result requires further verification. Figure 6 shows the main mechanistic pathways described in *in vivo /in vitro* experiments. The most important factors and pathways for Cd elevation and the pathogenesis of BC are as follows: ER, GPER, ROS, p53, PLP2, and β-catenin, respectively. ① Cd can increase BC cell proliferation by binding with ERα, which can then activate Akt, ERK, and PTK (PDGFRα/Src) kinases (142, 143). ② In ER-negative BC cells, Cd induces the activation of MEK/ERK through GPER, leading to the breed of BC cells (144). ③ Cd induces an increase in ROS concentration, and if persistent, this can lead to changes in oxide-reducing signaling pathways, DNA damages, and methylation and chromatin remodeling patterns (145). ④ Cd induces the increase of p53 in the cytoplasm by downregulating the expression of the p53 inhibitor, Ube2d, and ubiquitin-binding enzyme (146). ⑤ Cd, as a transcription regulator, upregulates the expression of PLP2, which encodes proteolipid protein 2 independently (147). ⑥ Cd also stimulates metastasis-related phenotypes of triple-negative BC cells through the activation of β-catenin signaling transduction (148). In hair and tissue, Cd concentration was not significantly different between cases and non-BC participants. Among the human specimens, blood Cd measurement could reflect not only long-term exposure but also short-term exposure (149), whereas hair content of Cd can be altered by dyeing, bleaching, and shampoos; further, the exposure concentration detected in hair depends on the distance from the scalp (129). The insufficient number of studies on Cd in tissue is another possible reason for the variation in findings.

#### Lead and Breast Cancer

Pb is another one of the potentially toxic trace elements for the general population. It gets into the human body mainly *via* the respiratory and digestive tract (150), but organic Pb compounds can also be absorbed by the skin in rare cases (151). More than 90% of Pb binds to erythrocytes in the form of Pb^2+^, once it enters the bloodstream (152). It can interplay with proteins in plasma and cellular, mostly the ones with thiol and sulfhydryl-containing (153). Pb is mostly discharged in the feces and urine (154). In this meta-analysis, there was no significant difference in plasma or serum Pb concentration between cases and non-BC participants; in hair, the concentration of Pb in patients with BC was significantly inferior to that in the control group. However, the number of studies included and the total sample size were comparatively small; thus, larger sample studies are required to confirm the relevance between Pb and BC.

In addition, studies on the mechanism concerning Pb in BC are limited, but the main research results show that Pb is tightly correlated with the pathogenesis of BC. Figure 6 shows the major mechanistic pathways from *in vivo / in vitro* experiments. The most important factor and pathway for Pb elevation and the pathogenesis of BC is ER signaling. Pb can activate ERα to direct the estrogen target genes expression and the BC cell reproduction (116). Moreover, Pb is a nonessential metal that can imitate or obstruct the function of essential metals to induce toxicity associated with BC (155, 156).

This study has several advantages. This is the first study to analyze the probable categories of heavy metals—essential or probably essential or potentially toxic—in patients with BC. It summarized the correlation using population studies data and mechanisms in detail. In addition, the conclusions were derived from different samples and regions. Besides, the publication bias was not found in the studies, meaning that these with obverse and the reverse results have been published, except the ones about Zn and Cd. However, there are also several limitations. First, terms of SMD were calculated to present the final pooled outcomes, which can eliminate the effect of multiple dimensions for lack of data measured in the uniformed dimension. However, the results in terms of SMD can only show whether there is a difference in heavy metal concentration between abnormal patients and non-BC participants. In the future, WMD can be used in analysis to get more information in clinical applications, if more original studies are conducted using data with the uniformed dimension. Second, this meta-analysis has included the studies on the relationship between heavy metals and BC comprehensively up to now. Although the quality of the studies has been evaluated with 5 or more points, several unmatched case–control studies included could partly lead to bias. Third, the results should be interpreted cautiously for possible publication bias about Zn and Cd. Fourth, it still cannot be analyzed about the effect of heavy metals on different degrees and molecular types of BC for the lack of related data, which are still needed to be studied further. Fifth, we summarized how Cr, Co, and Fe play roles in the mechanism of BC, but until now, there is only one population study on hair and tissue. Therefore, it remains to be verified in more population-based studies. Sixth, although the review and meta-analysis predicted the trends and also found the relative mechanism of effect of heavy metals in BC, heterogeneity in this meta-analysis was still not lowered after subgroup analysis, which may affect the stability of the results, but the mechanisms of heavy metals in BC had been found by signal pathways. Besides, although the differences in heavy metals in different biological specimens between patients with BC and patients with non-breast cancer are reviewed, whether heavy metals can directly lead to the incidence rate of BC cannot be verified. Therefore, the causal relationship requires more evidence in the future, and more prospective studies are still needed. In addition, the concentration of heavy metals in various biological samples has many factors (157). For example, hair Zn concentration is influenced by age, gender, season, hair growth rate, severity of malnutrition, and possibly hair color and other hair cosmetics (158). The concentrations of Cd, Pb in blood, and Pb in the hair seem to increase with smoking (159). In clinical application, the discovery of biomarkers of heavy metal contents with sensitivity and specificity in the prevention and diagnosis of diseases in the future is still needed.

The transport process of heavy metals is regulated by specific transporters. Divalent metal transporter 1 (DMT1), known as natural resistance-associated macrophage protein 2 (NRAMP2), divalent cation transporter 1 (DCT1), or solute carrier family 11, member 2 (SLC11A2), is a divalent metal transporter belonging to the proton-coupled metal-ion transporter family (160, 161). It regulates the transportation of bivalent metals including Fe, Zn, Mn, Cu, Co, Ni, Cd, and Pb (160). The increased expression of DMT1 could raise the uptake of Fe, Pb, and Cd in the duodenum (162, 163). Besides, DMT1 IVS4 + 44 C/A polymorphism impacts the individual differences in blood Fe, Pb, and Cd concentrations (161). However, few pieces of evidence have been reported relating to the ethnic and genetic differences in the transport, absorption, and elimination of heavy metals, which are required to be further studied.

## Conclusion

On the whole, the findings of this study made clear that heavy metals may be related to BC. In terms of the essential trace elements, a higher concentration of Cu and a lower concentration of Zn in plasma/serum were observed in patients with BC as compared to patients with non-BC. For the probably essential trace elements, higher Mn in plasma/serum may help to reduce the BC risk. For the potentially toxic trace elements, higher Cd may be associated with the BC risk. In hair, we only observed a beneficial effect of Zn on BC development. No significant differences were indicated in plasma or serum samples of Cr, Co, and Ni between cases and non-BC participants. For the higher elements, studies with larger sample sizes and in various populations are needed to further verify the roles of these heavy metals in BC. To sum up, different from the ordinary population, the role of trace elements probably needs to be re-explored, particularly in BC.

## Data Availability Statement

The original contributions presented in the study are included in the article/Supplementary Material, further inquiries can be directed to the corresponding authors.

## Author Contributions

XL designed, supervised the study, guided research methods, and revised the manuscript. LL and JieC extracted the data, collected, analyzed, and double checked the data. LL wrote and revised the manuscript. LL, JieC, CL, YL, JiaC, YF, YX, HWu, and HWa reviewed and edited the manuscript. All authors have read and approved the final draft.

## Funding

This study was supported by the grants from the Scientific research project of the Shanghai Health Committee (201940188); the Three-Year Action Program of the Shanghai Municipality for Strengthening the Construction of Public Health System (GWV-10.2-YQ34, GWV-10.1-XK05); the National Key R&D Program of China (2018YFC2000700); the SJTU Public Health School Local High-Level University Achievement-oriented Top-Notch Cultivation Program for Undergraduate Students (18ZYGW06); the innovative research team of high-level local universities in Shanghai.

## Conflict of Interest

The authors declare that the research was conducted in the absence of any commercial or financial relationships that could be construed as a potential conflict of interest. The reviewer JL declared a shared affiliation with the authors at the time of review.

## Publisher's Note

All claims expressed in this article are solely those of the authors and do not necessarily represent those of their affiliated organizations, or those of the publisher, the editors and the reviewers. Any product that may be evaluated in this article, or claim that may be made by its manufacturer, is not guaranteed or endorsed by the publisher.
