# Supplementary material for: Relationships Between Biological Heavy Metals and Breast Cancer: A Systematic Review and Meta-Analysis
Source: Front Nutr. 2022 Jun 6;9:838762. doi: 10.3389/fnut.2022.838762 (PMC9245072; doi:10.3389/fnut.2022.838762)
Supplement: Supplementary file 1 [file Table_1.DOCX]

Supplementary Material

**Supplementary table 1. Quality assessment of included articles using Newcastle-Ottawa scale (NOS).**

| **Study** | **Country** | **Selection** | | | | **Comparability** | | **Exposure** | | | **Total** |
| --- | --- | --- | --- | --- | --- | --- | --- | --- | --- | --- | --- |
|  |  | **1** | **2** | **3** | **4** | **1** | **2** | **1** | **2** | **3** |  |
| C M Tinoco-Veras 2011 | Brazil | * | * |  | * |  |  | * | * |  | ***** |
| Camila Guedes Borges de Araújo 2015 | Brazil | * | * |  | * |  |  | * | * |  | ***** |
| I D Capel 1982 | England | * | * |  | * |  |  | * | * |  | ***** |
| V Singh 1997 | India | * | * |  | * |  |  | * | * |  | ***** |
| M K J Siddiqui 2006 | India | * | * |  | * | * |  | * | * |  | ****** |
| Deepti Pande 2013 | India | * | * |  | * | * |  | * | * |  | ****** |
| V Pavithra 2015 | India | * | * |  | * | * |  | * | * |  | ****** |
| S K Gupta 1991 | India | * | * |  | * | * |  | * | * |  | ****** |
| Kanchan Karki 2015 | India | * | * |  | * | * |  | * | * |  | ****** |
| P. Sarita 2012 | India | * | * |  | * | * |  | * | * |  | ****** |
| N Gholizadeh 2013 | Iran | * | * |  | * |  |  | * | * |  | ***** |
| Asghar Maziar 2015 | Iran | * | * |  | * |  |  | * | * |  | ***** |
| F Cavallo 1991 | Italy/France | * | * |  | * | * |  | * | * |  | ****** |
| L Piccinini 1996 | Italy | * | * |  | * | * | * | * | * |  | ******* |
| Nam-Seok Joo 2009 | Korea | * | * |  | * | * | * | * | * |  | ******* |
| Farid Saleh 2011 | Kuwait | * | * |  | * | * |  | * | * |  | ****** |
| M L Adeoti 2015 | Nigeria | * | * |  | * | * |  | * | * |  | ****** |
| G O Ajayi 2011 | Nigeria | * | * |  | * |  |  | * | * |  | ***** |
| Sham Kumar Wadhwa 2015 | Pakistan | * | * |  | * |  |  | * | * |  | ***** |
| M Zowczak 2001 | Poland | * | * |  | * |  |  | * | * |  | ***** |
| Yasemin Benderli Cihan 2011 | Turkey | * | * |  | * | * |  | * | * |  | ****** |
| Eser Kilic 2004 | Turkey | * | * |  | * | * |  | * | * |  | ****** |
| I Yücel 1994 | Turkey | * | * |  | * |  |  | * | * |  | ***** |
| C Köksoy 1997 | Turkey | * | * |  | * | * |  | * | * |  | ****** |
| C Yenisey 1996 | Turkey | * | * |  | * |  |  | * | * |  | ***** |
| M Garland 1996 | USA | * | * |  | * |  |  | * | * |  | ***** |
| K Overvad 1993 | England | * | * |  | * | * |  | * | * |  | ****** |
| K Geraki 2002 | England | * | * |  | * |  |  | * | * |  | ***** |
| E Antila 1996 | Finland | * | * |  | * |  |  | * | * |  | ***** |
| E J Margalioth 1983 | Israel | * | * |  | * |  |  | * | * |  | ***** |
| P..A.I. Ismail 2017 | Iraq | * | * |  | * |  |  | * | * |  | ***** |
| Luhua Wang 2015 | China | * | * |  | * | * |  | * | * |  | ****** |
| Dong Wang 2012 | China | * | * |  | * | * |  | * | * |  | ****** |
| Te-Hsien Lin 1999 | China | * | * |  | * | * |  | * | * |  | ****** |
| Hsien-Wen Kuo 2006 | China | * | * |  | * |  |  | * | * |  | ***** |
| C.T.Wang 2006 | China | * | * |  | * | * |  | * | * |  | ****** |

**Supplementary Table 2.** **Quality assessment of included articles using Critical Appraisal Skills Programme (CASP) checklist.**

| **Study** | **Focused question** | **Appropriate method to answer the question** | **Recruitment of cases** | **Recruitment of controls** | **Exposure accurately measured** | **Aside from the experimental intervention, were the groups treated equally** | **Confounding factors taken into account in the design/analysis** | **What are the results?** | **How precise are the results?** | **Do you believe the results?** | **Can the results be applied to the local population?** | **Do the results of this study fit with other available evidence?** |
| --- | --- | --- | --- | --- | --- | --- | --- | --- | --- | --- | --- | --- |
| C M Tinoco-Veras 2011 | Yes | Yes, case- control design. | Hospital based. | No mention | Plasma Zn concentration was measured by atomic absorption  spectrophotometry. | No mention. | Excluded women in use of medication or vitamin  / mineral supplements and  those with acute or chronic diseases that could affect normal Zn metabolism. | The mean plasma Zn concentration were  higher in BC. | Results are precise. | Yes | Internal validity Yes, there may be variabilities across different endemicity regions. | Generalizability, yes. |
| Camila Guedes Borges de Araújo 2015 | Yes | Yes, case- control design | No mention | No mention | Plasma Zn concentration was determined by atomic absorption spectrophotometry. | Control group consisted of female, premenopausal  volunteers with no breast cancer diagnosis, FSH not  greater than 30 μg/mL, no chronic diseases and no prior history of treatment for the disease | Women with serum levels  of follicle stimulating hormone (FSH) > 30 μg/mL and  those with chronic diseases such as diabetes and hypertension  and history of previous treatment for the disease  and patients using medications  and vitamin / mineral supplements that could interfere with zinc metabolism were excluded. | The mean plasma Zn concentration were  Lower in BC. | Results are precise. | Yes | Internal validity Yes, there may be variabilities across different endemicity regions. | Generalizability, yes. |
| I D Capel 1982 | Yes | Yes, case- control design | Hospital based. | Control selected from different hospital. | Plasma Cu, Zn, Mn concentration were determined by atomic absorption spectrophotometry. | Control patients were 10 geriatric in-patients with non-malignant conditions at St. Pancras Hospital. | Recent post-operative patients (within 2 weeks) and subjects in whom metastases were suspected but not confirmed were excluded. | The serum Cu levels were higher in BC. | Results are precise. | Yes | Internal validity Yes, there may be variabilities across different endemicity regions. | Generalizability, yes. |
| V Singh 1997 | Yes | Yes, case- control design | Hospital based. | No mention | Plasma Zn, Fe, Co, Cr concentration were determined. | No mention | No mention | The study showed elevation of Cr, Co, Fe and Zn in the blood of cases. | Results are precise. | Yes | Internal validity Yes, there may be variabilities across different endemicity regions. | Generalizability, yes. |
| M K J Siddiqui 2006 | Yes | Yes, case- control design | Hospital based. | Control selected from same hospital. | Plasma Cu, Zn, Fe, Pb concentration were determined by flame atomic absorption spectrometer and graphite furnace atomic absorption  spectrometer. | Age matched between cases and controls. | Excluded the patients who had a mamographically detected non-palpable lesion or a lump b 1 cm in size. | The study showed elevation of Pb, Fe and Zn in the blood of cases. | Results are precise. | Yes | Internal validity Yes, there may be variabilities across different endemicity regions. | Generalizability, yes. |
| Deepti Pande 2013 | Yes | Yes, case- control design | No mention | No mention | Plasma Cu, Zn, Fe concentration were determined. | Age, sex and socioeconomic matched. | Excluded  patients  with clinical or pathological evidence of cancer at any other  site or of having received any type of neo-adjuvant therapy  along with the presence of liver dysfunction, diabetes mellitus,  heart or renal failure. | The study showed elevation of Cu and Zn in the blood of cases. | Results are precise. | Yes | Internal validity Yes, there may be variabilities across different endemicity regions. | Generalizability, yes. |
| V Pavithra 2015 | Yes | Yes, case- control design | Hospital based. | Control selected from same hospital. | Plasma Cu, Zn, Fe concentration were determined. | Age matched. | The patients or controls suffering from co-morbid conditions which affect serum levels of metal ions and other malignancies, and/or undergoing treatment for breast cancer were excluded. | The study showed elevation of Cu and Fe and decline of Zn in the blood of cases. | Results are precise. | Yes | Internal validity Yes, there may be variabilities across different endemicity regions. | Generalizability, yes. |
| S K Gupta 1991 | Yes | Yes, case- control design | No mention | No mention | Plasma Cu, Zn concentration were determined by atomic absorption spectrophotometer. | Age matched. | No mention | The study showed elevation of Cu and decline of Zn in the blood of cases. | Results are precise. | Yes | Internal validity Yes, there may be variabilities across different endemicity regions. | Generalizability, yes. |
| Kanchan Karki 2015 | Yes | Yes, case- control design | Hospital based. | Control selected from same hospital. | Plasma Cu, Zn, Fe concentration were determined. | Age and sex matched. | The healthy controls having no acute or chronic diseases such as diabetes, parasitosis, immune dysfunction or any other malignancy and they were not under any pharmacological therapy. None of the study subjects were under oral contraceptives, hormonal therapy or antioxidant supplementation. | Decreased levels of Cu, Zn and Fe in cases. | Results are precise. | Yes | Internal validity Yes, there may be variabilities across different endemicity regions. | Generalizability, yes. |
| P. Sarita 2012 | Yes | Yes, case- control design | No mention | No mention | Plasma Cu, Zn, Fe, Mn, Ni, Co, Cr concentration were determined by PIXE. | Age, sex and place of residence  matched. | No mention | The study showed elevation of Cu and Fe decline of Zn, Mn, Ni, Co and Cr in the blood of cases. | Results are precise. | Yes | Internal validity Yes, there may be variabilities across different endemicity regions. | Generalizability, yes. |
| N Gholizadeh 2013 | Yes | Yes, case- control design | Hospital based. | Control selected from same hospital. | Hair Cu, Zn, Fe concentration were determined by PIXE. | No mention | No mention | Decreased levels of Zn in cases. No significant difference in Fe and Cu. | Results are precise. | Yes | Internal validity Yes, there may be variabilities across different endemicity regions. | Generalizability, yes. |
| Asghar Maziar 2015 | Yes | Yes, case- control design | Hospital based. | Control selected from same hospital. | Hair Zn concentration were determined by XRF. | No mention | The hair sam­ples were obtained from women who had no colored hair for 6 weeks or more and hair samples of cancer cases were collected from patients who had not undertaken chemotherapy and medicine therapy. Hair strands were cut from occipital and parietal region. | Zn in controls was higher than cases. | Results are precise. | Yes | Internal validity Yes, there may be variabilities across different endemicity regions. | Generalizability, yes. |
| F Cavallo 1991 | Yes | Yes, case- control design | Hospital based. | Control selected from different hospital. | Serum Cu, Zn concentration were determined by atomic absorption spectrophotometry. | Region matched. | No mention | Zn in cases was higher than controls. Cu blood level showed higher in controls in Milan but higher in cases in Montpellier. | Results are precise. | Yes | Internal validity Yes, there may be variabilities across different endemicity regions. | Generalizability, yes. |
| L Piccinini 1996 | Yes | Yes, case- control design | No mention | No mention | Plasma/hair Cu, Zn concentration were determined by atomic absorption  Spectrometry. | Sex, age, smoking habits, and residence matched. | No mention | No difference  between cases and controls was detected in both hair and plasma levels  of Zn and Cu. | Results are precise. | Yes | Internal validity Yes, there may be variabilities across different endemicity regions. | Generalizability, yes. |
| Nam-Seok Joo 2009 | Yes | Yes, case- control design | Hospital based. | Hospital based. | Hair Cu, Zn, Fe, Cd, Mn, Pb, Cr concentration were determined. | Age and BMI matched. | Cases had only breast cancer without any other cancer. | Breast cancer patients had low Fe, Cu, Mn, and Zn. | Results are precise. | Yes | Internal validity Yes, there may be variabilities across different endemicity regions. | Generalizability, yes. |
| Farid Saleh 2011 | Yes | Yes, case- control design | No mention | No mention | Serum Cu, Zn, Cd concentration were determined by atomic absorption  spectrophotometer. | Age matched. | No mention | Breast cancer patients had lower Cu, Zn and higher  Cd. | Results are precise. | Yes | Internal validity Yes, there may be variabilities across different endemicity regions. | Generalizability, yes. |
| M L Adeoti 2015 | Yes | Yes, case- control design | Hospital based. | Hospital based. | Serum Cu and Zn concentration were determined by atomic absorption  Spectrophotometer. | Age and sex matched. | Male patients, patients on vitamin or mineral supplements,  patients diagnosed of other co‑morbidities including infective  diseases and patients who were pregnant or  lactating, patients who have had any form of treatment for their disease and patients on hormone replacement therapy or oral contraceptives were excluded. | Breast cancer patients had higher Cu and lower Zn. | Results are precise. | Yes | Internal validity Yes, there may be variabilities across different endemicity regions. | Generalizability, yes. |
| G O Ajayi 2011 | Yes | Yes, case- control design | No mention | No mention | Serum Cu and Zn concentration were determined by atomic absorption  spectrophotometer. | Age matched. | No mention | Breast cancer patients had higher Cu and lower Zn. | Results are precise. | Yes | Internal validity Yes, there may be variabilities across different endemicity regions. | Generalizability, yes. |
| Sham Kumar Wadhwa 2015 | Yes | Yes, case- control design | Hospital based. | They  Were mostly the healthy family members of the patients. | Hair Zn, Cd and Ni concentration were determined by atomic absorption  spectrophotometer. | Age, socio-economic status and dietary habits matched. | Female not suffering  from any cancerous lesions and taking any mineral supplement. | Breast cancer patients had higher Ni, Cd and lower Zn. | Results are precise. | Yes | Internal validity Yes, there may be variabilities across different endemicity regions. | Generalizability, yes. |
| M Zowczak 2001 | Yes | Yes, case- control design | Hospital based. | Hospital based. | Serum Cu and Zn concentration were determined by flame atomic absorption spectrometer. | Age and sex matched. | No mention | Breast cancer patients had higher Cu. | Results are precise. | Yes | Internal validity Yes, there may be variabilities across different endemicity regions. | Generalizability, yes. |
| Yasemin Benderli Cihan 2011 | Yes | Yes, case- control design | Hospital based. | Control selected from same hospital. | Hair Cu, Zn, Fe, Cd, Mn, Pb, Ni, Co and Cr concentration were determined by ICP/MS. | Age matched. | Patients at other stages were excluded as well as those with recurrent  breast cancer, rheumatoid arthritis or inflammatory disease, diabetes, hyperthyroidism,  hypertension, secondary cancer, dyed or cosmetically altered hair, received vitamins or  mineral supplements for 1 year, and male patients. | Breast cancer patients had higher Cd, Co and lower Ni, Pb Zn. | Results are precise. | Yes | Internal validity Yes, there may be variabilities across different endemicity regions. | Generalizability, yes. |
| Eser Kilic 2004 | Yes | Yes, case- control design | Hospital based. | Control selected from same hospital. | Hair Mn and Cr concentration were determined by graphite furnace atomic absorption. | Age matched. | Samples that had suspiciously high concentrations of  Ag, Ni, and Pb, typical for external contaminations because of hair treatment  procedures were excluded. | Breast cancer patients had higher Cr and lower Mn. | Results are precise. | Yes | Internal validity Yes, there may be variabilities across different endemicity regions. | Generalizability, yes. |
| I Yücel 1994 | Yes | Yes, case- control design | No mention | No mention | Serum Cu and Zn concentration were determined by atomic absorption spectrophotometry. | Age matched. | None of the  patients were using hormones, oral contraceptives, or Cu or Zn  supplements. No infection was observed in any patient during the study.  No significant weight loss, impaired appetite, or change in eating patterns  were detected in the patients. | Breast cancer patients had higher Cr and lower Mn. | Results are precise. | Yes | Internal validity Yes, there may be variabilities across different endemicity regions. | Generalizability, yes. |
| C Köksoy 1997 | Yes | Yes, case- control design | Hospital based. | Control selected from same hospital. | Plasma Cu and Zn concentration were determined by atomic absorption  spectrophotometry. | Age matched. | No mention | Breast cancer patients had higher Cu. | Results are precise. | Yes | Internal validity Yes, there may be variabilities across different endemicity regions. | Generalizability, yes. |
| C Yenisey 1996 | Yes | Yes, case- control design | Hospital based. | Control selected from same hospital. | Serum Cu concentration was determined. | No mention | No mention | Breast cancer patients had higher Cu. | Results are precise. | Yes | Internal validity Yes, there may be variabilities across different endemicity regions. | Generalizability, yes. |
| M Garland 1996 | Yes | Yes, case- control design | No mention | No mention | Toenail Cu, Zn, Fe and Cr concentration were determined by instrumental neutron activation analysis. | Year of birth and month of nail return (the nails were returned over a  15-month period) match. | Excluded their date of nail  return was after their date of diagnosis or their diagnosis  was rejected based on the medical record  review. | There were no significant differences in Cu, Zn, Fe and Cr between cases and controls. | Results are precise. | Yes | Internal validity Yes, there may be variabilities across different endemicity regions. | Generalizability, yes. |
| K Overvad 1993 | Yes | Yes, case- control design | No mention | No mention | Plasma Cu concentration was determined by PIXE. | Age matched. | No mention | Breast cancer patients had higher Cu. | Results are precise. | Yes | Internal validity Yes, there may be variabilities across different endemicity regions. | Generalizability, yes. |
| K Geraki 2002 | Yes | Yes, case- control design | Hospital based. | Hospital based. | Tissue Cu, Zn and Fe concentration were determined by x-ray fluorescence. | No mention | No mention | Breast cancer patients had higher Cu, Fe and Zn. | Results are precise. | Yes | Internal validity Yes, there may be variabilities across different endemicity regions. | Generalizability, yes. |
| E Antila 1996 | Yes | Yes, case- control design | Hospital based. | Samples were taken  during routine postmortem examinations  from accidental fatalities or women  who died of a sudden nonmalignant illness. | Tissue Cd concentration was determined by atomic absorption spectrophotometry. | Age, height, and weight matched. | No mention | There were no significant differences in Cd between cases and controls. | Results are precise. | Yes | Internal validity Yes, there may be variabilities across different endemicity regions. | Generalizability, yes. |
| E J Margalioth 1983 | Yes | Yes, case- control design | Hospital based. | Hospital based. | Tissue Cu and Zn concentration were determined by absorption spectrophotometry. | No mention | No mention | Breast cancer patients had higher Zn. | Results are precise. | Yes | Internal validity Yes, there may be variabilities across different endemicity regions. | Generalizability, yes. |
| P..A.I. Ismail 2017 | Yes | Yes, case- control design | No mention | No mention | Serum Fe, Cd, Ni, Cr, Mn, Co concentration were determined by absorption spectrophotometry. | No mention | No mention | Serum  Cd, Ni, Cr, Fe and Co were higher,  serum Mn was lower  in cases. | Results are precise. | Yes | Internal validity Yes, there may be variabilities across different endemicity regions. | Generalizability, yes. |
| Luhua Wang 2015 | Yes | Yes, case- control design | Hospital based. | Control selected from same hospital. | Serum Cu, Zn, Fe, Cd, Mn, Pb, Ni, Co and Cr concentration were determined by atomic emission spectrometer. | Age, residence  and other established risk factors of breast cancer matched. | Excluded those diagnosed of any malignancy  other than primary breast cancer. | Breast cancer patients had higher Cd, Cu, Co and lower Mn, Fe. | Results are precise. | Yes | Internal validity Yes, there may be variabilities across different endemicity regions. | Generalizability, yes. |
| Dong Wang 2012 | Yes | Yes, case- control design | Hospital based. | Control selected from same hospital. | Serum Cu, Zn, Fe and Mn concentration were determined by atomic absorption spectroscopy / flame atomic absorption  Spectroscopy / graphite furnace atomic  absorption spectroscopy. | Age matched. | Patients with other diseases, such as  diabetes mellitus, hypoglycemia, gout, protein-energy  malnutrition, vitamin A/D deficiency, thyroid disease,  osteoporosis, rheumatoid arthritis, and/or liver diseases,  were excluded. | Breast cancer patients had higher Cu, Fe and lower Zn, Mn. | Results are precise. | Yes | Internal validity Yes, there may be variabilities across different endemicity regions. | Generalizability, yes. |
| Te-Hsien Lin 1999 | Yes | Yes, case- control design | No mention | No mention | Serum Cu, Zn and Fe concentration were determined by flame atomic absorption spectroscopy. | Age, geographical area and socio-sanitary situation matched. | None of the persons were using  hormones, oral contraceptives, or trace elements containing  supplements. All were nonsmokers.  Patients  with concomitant diseases such as diabetes mellitus,  rheumatoid arthritis, Wilson’s disease, or liver disorders  were excluded. | Breast cancer patients had higher Cu. | Results are precise. | Yes | Internal validity Yes, there may be variabilities across different endemicity regions. | Generalizability, yes. |
| Hsien-Wen Kuo 2006 | Yes | Yes, case- control design | Hospital based. | Hospital based from the same region. | Serum Cu, Zn, Fe, Cd, Mn, Ni, Co and Cr concentration were determined by atomic emission spectrometry and atomic absorption spectrophotometry. | No mention | No mention | Breast cancer patients had higher Cd, Ni, Fe, Cr, Cu and lower Zn, Mn. | Results are precise. | Yes | Internal validity Yes, there may be variabilities across different endemicity regions. | Generalizability, yes. |
| C.T.Wang 2006 | Yes | Yes, case- control design | Hospital based. | Control selected from same hospital. | Hair Cu, Zn and Fe concentration were determined by atomic absorption spectrophotometry. | Age matched. | Hair was cut near the scalp area  with thin-blade stainless steel scissors. The  average length of hair ranged 1.0-3.0 cm. Hair was weighed to 1.000 ± 0.200 g. | Breast cancer patients had higher Fe, Cu and lower Zn. | Results are precise. | Yes | Internal validity Yes, there may be variabilities across different endemicity regions. | Generalizability, yes. |

**Supplementary Table 3.** **Characteristics of the researches used for the meta-analysis.**

| **Study** | **Continent** | **Age (years): mean ± SD/mean (range)** | **Species** | **Measurement** | **BC** | **Non-BC** | **Heavy metals** |
| --- | --- | --- | --- | --- | --- | --- | --- |
| G O Ajayi 2011 | Africa | No mention | Serum | AAS | 11 | 9 | Cu、Zn |
| M L Adeoti 2015 | Africa | case:47.3±6.8; control:46.5±6.8 | Serum | AAS | 30 | 30 | Cu、Zn |
| C.T.Wang 2006 | Asia | case:54.2 ± 12.9; control: 52.2 ± 12.2 | Hair | AAS | 50 | 50 | Cu、Zn、Fe |
| Dong Wang 2012 | Asia | case:48.7±8.4;  control: 47.2±7.6 | Serum | AAS/FAAS  /GFAAS | 56 | 20 | Cu、Zn、Fe、Mn |
| Hsien-Wen Kuo 2006 | Asia | No mention | Serum | AES/AAS | 25 | 26 | Cu、Zn、Fe、Cd、Mn、Ni、Co、Cr |
| Luhua Wang 2015 | Asia | case:45.73±9.84;  control: 45.29±8.52 | Serum | AES | 88 | 84 | Cu、Zn、Fe、Cd、Mn、Pb、Ni、Co、Cr |
| Te-Hsien Lin 1999 | Asia | case:48.2±12.1;  control: 44.5±10.2 | Serum | FAAS | 35 | 35 | Cu、Zn、Fe |
| Deepti Pande 2013 | Asia | 46.5 | Serum | No mention | 40 | 40 | Cu、Zn、Fe |
| Kanchan Karki 2015 | Asia | No mention | Serum | No mention | 70 | 70 | Cu、Zn、Fe |
| M K J Siddiqui 2006 | Asia | 32-60  (case:46.84±6.90; control:42.37±12.31) | Serum | GFAAS/FAAS | 25 | 25 | Cu、Zn、Fe、Pb |
| P. Sarita 2012 | Asia | 25-75 | Serum | PIXE | 21 | 30 | Cu、Zn、Fe、Mn、Ni、Co、Cr |
| S K Gupta 1991 | Asia | case:47± 9; control:46±10 | Serum | AAS | 35 | 30 | Cu、Zn |
| V Pavithra 2015 | Asia | case:47.2±8.14; control:46.8±8.4 | Serum | No mention | 54 | 54 | Cu、Zn、Fe |
| V Singh 1997 | Asia | 30-60 | Serum | No mention | 10 | 5 | Zn、Fe、Co、Cr |
| Asghar Maziar 2015 | Asia | 33-79  (52.03 ± 11.44) | Hair | XRF | 27 | 27 | Zn |
| N Gholizadeh 2013 | Asia | 22-70 | Hair | PIXE | 21 | 10 | Cu、Zn、Fe |
| E J Margalioth 1983 | Asia | No mention | Tissue | AAS | 8 | 4 | Cu、Zn |
| Nam-Seok Joo 2009 | Asia | case:47.1± 9.6; control:47.8±5.1 | Hair | No mention | 40 | 144 | Cu、Zn、Fe、Cd、Mn、Pb、Cr |
| Farid Saleh 2011 | Asia | case:47.2± 11.77; control:46.9±12.52 | Serum | AAS | 50 | 150 | Cu、Zn、Cd |
| Sham Kumar Wadhwa 2015 | Asia | No mention | Hair | AAS | 47 | 94 | Zn、Cd、Ni |
| P..A.I. Ismail 2017 | Asia | No mention | Serum | AAS | 40 | 40 | Fe, Cd, Ni, Cr, Mn, Co |
| I D Capel 1982 | Europe | 52-81 | Serum | AAS | 52 | 10 | Cu、Zn、Mn |
| K Overvad 1993 | Europe | case:45.2± 10; control:44.7±10.4 | Plasma | PIXE | 46 | 138 | Cu |
| K Geraki 2002 | Europe | No mention | Tissue | XRF | 20 | 20 | Cu、Zn、Fe |
| E Antila 1996 | Europe | average  (case: 56; control: 33) | Tissue | AAS | 43 | 32 | Cd |
| F Cavallo 1991-B | Europe | case:53.1 ±9.8; control:53.1 ±8.6 | Serum | AAS | 47 | 46 | Cu、Zn |
| L Piccinini 1996 | Europe | 41-79  (case:58.6±10.3; control:56.8±11) | Plasma  /hair | FAAS | 38 | 22 | Cu、Zn |
| F Cavallo 1991-A | Europe | case:49.6 ±8.5; control:47.1 ± 9.4 | Serum | AAS | 207 | 212 | Cu、Zn |
| M Zowczak 2001 | Europe | case:50-70; control:20-40 | Serum | FAAS | 8 | 21 | Cu、Zn |
| Yasemin Benderli Cihan 2011 | Europe | case:50.3±8.8; control:47.4±10.1 | Hair | ICP/MS | 52 | 52 | Cu、Zn、Fe、Cd、Mn、Pb、Ni、Co、Cr |
| Eser Kilic 2004 | Europe | case:53±9;  control:55±7 | Hair | GFAAS | 26 | 27 | Mn、Cr |
| I Yücel 1994 | Europe | case:28-75;  control:35-80 | Serum | AAS | 31 | 35 | Cu、Zn |
| C Köksoy 1997 | Europe | No mention | Plasma | AAS | 39 | 20 | Cu、Zn |
| C Yenisey 1996 | Europe | case:30-70;  control:18-52 | Serum | No mention | 108 | 26 | Cu |
| M Garland 1996 | North America | 30-55 | Toenail | INAA | 433 | 459 | Cu、Zn、Fe、Cr |
| C M Tinoco-Veras 2011 | South America | 25-49 | Plasma | FAAS | 29 | 26 | Zn |
| Camila Guedes Borges de Araújo 2015 | South America | 25-50 | Plasma | AAS | 34 | 32 | Zn |

AAS atomic absorption spectrometry, AES atomic emission spectrometry, GFAAS graphite furnace atomic absorption spectrometry, PIXE proton-induced X-ray emission, FAAS Flame atomic absorption spectrometry, MS mass spectrometry, CP catalytic polarography, ICP inductively coupled plasma, XRF X-ray fluorescence spectrometry, INAA instrumental neutron activation analysis

**Supplementary Table 4.** **The association between heavy metals and** **breast cancer in meta-analysis.**

| **Category** | **Heavy metal** | **Species** | **No. of studies** | **SMD (95% CIs)** | ***P-*value** | ***I^2^*** | **Egger test** |
| --- | --- | --- | --- | --- | --- | --- | --- |
| essential trace elements | Cu | all | 30 | 0.62(0.12, 1.12) | 0.01 | 97% | 0.21 |
|  |  | plasma and serum | 22 | 0.98(0.36, 1.60) | 0.002 | 97% |  |
|  |  | hair | 5 | 0.96(-3.15, 1.23) | 0.39 | 99% |  |
|  |  | toenails | 1 | -0.02(-0.15, 0.11) | 0.80 | - |  |
|  |  | tissue | 2 | 1.24(0.06, 2.41) | 0.04 | 64% |  |
|  | Zn | all | 33 | -1.40(-1.96, -0.85) | <0.0001 | 98% | 0.0053 |
|  |  | plasma and serum | 23 | -1.53(-2.28, -0.78) | <0.0001 | 98% |  |
|  |  | hair | 7 | -2.12(-3.55, -0.68) | 0.004 | 98% |  |
|  |  | toenails | 1 | -0.02(-0.15, 0.11) | 0.76 | - |  |
|  |  | tissue | 2 | 1.50(0.86,2.13) | <0.00001 | 0% |  |
|  | Cr | all | 9 | 0.71(-0.21, 1.63) | 0.13 | 98% | 0.46 |
|  |  | plasma and serum | 5 | 1.48(-0.02, 3.17) | 0.08 | 97% |  |
|  |  | hair | 3 | -0.23(-2.85, 2.40) | 0.86 | 99% |  |
|  |  | toenails | 1 | 0.01(-0.12, 0.15) | 0.83 | - |  |
|  | Co | all | 6 | 0.64(-0.53, 1.81) | 0.28 | 96% | 0.87 |
|  |  | plasma and serum | 5 | 0.61(-0.99, 2.21) | 0.45 | 97% |  |
|  |  | hair | 1 | 0.81(0.41, 1.21) | <0.0001 | - |  |
|  | Fe | all | 17 | 0.16(-0.71, 1.02) | 0.72 | 98% | 0.92 |
|  |  | plasma and serum | 11 | 0.96(-0.46, 2.38) | 0.19 | 98% |  |
|  |  | hair | 4 | -1.94(-4.68, 0.81) | 0.17 | 99% |  |
|  |  | toenails | 1 | -0.03(-0.16, 0.11) | 0.70 | - |  |
|  |  | tissue | 1 | 0.92(0.27,1.58) | 0.006 | - |  |
| probably essential trace elements | Ni | all | 6 | 1.21(-0.18, 2.59) | 0.09 | 98% | 0.14 |
|  |  | plasma and serum | 4 | 0.99(-0.64, 2.61) | 0.24 | 97% |  |
|  |  | hair | 2 | 1.65(-1.96, 5.27) | 0.37 | 99% |  |
|  | Mn | all | 9 | -2.26(-3.39, -1.13) | <0.0001 | 97% | 0.10 |
|  |  | plasma and serum | 6 | -2.40(-3.69, -1.10) | 0.0003 | 96% |  |
|  |  | hair | 3 | -1.99(-4.69, 0.70) | 0.15 | 99% |  |
| potentially toxic trace elements | Cd | all | 8 | 1.64(0.76, 2.52) | 0.0003 | 97% | 0.06 |
|  |  | plasma and serum | 4 | 2.55(1.16, 3.94) | 0.0003 | 97% |  |
|  |  | hair | 3 | 1.16(-0.38, 2.70) | 0.14 | 98% |  |
|  |  | tissue | 1 | -0.39(-0.85,0.08) | 0.20 | - |  |
|  | Pb | all | 4 | 2.03(0.11, 3.95) | 0.04 | 98% | 0.70 |
|  |  | plasma and serum | 2 | 3.94(-4.62, 12.50) | 0.37 | 99% |  |
|  |  | hair | 2 | -0.41(-0.80, -0.02) | 0.04 | - |  |

**Supplementary file 1. Systematic literature electronic search strategy.**

**Literature sources**

English language Studies published up until September 2020 were identified, through electronic searches using PubMed, Web of Science and Embase databases and discussion with investigators. The computer-based search strategy is detailed below. Upon identification of studies and eligible independent human case-control, cohort study or randomized clinical trials, 36 case-control studies were investigated to participate in a standardized individual participant data analysis of relationship between heavy metals and breast cancer by September 2020.

**Search strategy**

Publication database: PubMed

#1 “heavy metals” OR “trace elements”

#2 “breast cancer” OR “mammary carcinoma”

Search #1 AND #2

Restrictions case-control study, cohort study or randomized clinical trial

# identified studies 12128

Publication database: Web of Science

#1 (heavy metals OR trace elements)

#2 (breast cancer OR mammary carcinoma)

Search #1 AND #2

Restrictions case-control study, cohort study or randomized clinical trial

# identified studies 557

Publication database: Embase databases

#1 TITLE-ABS-KEY (heavy metals OR trace elements)

#2 TITLE-ABS-KEY (breast cancer OR mammary carcinoma)

Search #1 AND #2

Restrictions case-control study, cohort study or randomized clinical trial

# identified studies 139
